# A new *Antaeotricha* species from Florida sandhills and scrub (Lepidoptera, Depressariidae, Stenomatinae)

**DOI:** 10.3897/zookeys.533.6004

**Published:** 2015-11-09

**Authors:** James E. Hayden, Terhune S. Dickel

**Affiliations:** 1 Florida Department of Agriculture and Consumer Services, Division of Plant Industry, 1911 SW 34th Street, Gainesville, FL 32608 USA; 2 P.O. Box 567, Anthony, FL 32617 USA; Research Associate, Florida State Collection of Arthropods, Gainesville, FL, USA

**Keywords:** *Quercus geminata*, *Quercus minima*, sand pine scrub

## Abstract

*Antaeotricha floridella* **sp. n.** is described and diagnosed from the closely similar *Antaeotricha albulella* (Walker). The species is distributed in xeric sandhill and scrub habitats in peninsular Florida, USA, and larvae feed on *Quercus* species. Keys are given for pale-winged Stenomatinae and similar Gelechioidea based on external characters and genitalia.

## Introduction

The genus *Antaeotricha* Zeller, 1854 (Lepidoptera: Depressariidae: Stenomatinae) is endemic to the New World and includes nearly 400 species, mostly in the Neotropics (Becker 1984). Twenty Nearctic species are known (Ferris 2013), including the one described below. Duckworth (1964) comprehensively revised the Nearctic Stenomatinae, and four species were recently described from the southwestern United States (Ferris 2010, 2012, 2013).

The second author (TSD) discovered the presently described species by dissection, as it is externally very similar to the widely distributed *Antaeotricha albulella* (Walker, 1864) (more often called by its junior synonym *Antaeotricha vestalis* (Zeller, 1873) [Becker 1981]). The search of other collections by JEH yielded more specimens, including ones overlooked by Duckworth. The species description necessitates a revised key to pale-colored Nearctic Stenomatinae. The species is known only from xeric habitats in peninsular Florida. This pattern of endemism of species in Florida sandhills is common among other orders of insects and arthropods but is infrequent in Lepidoptera (Deyrup 1989), so the addition of another endemic species is significant.

Stenomatinae are diagnosed by the presence of thickened, apically bifid or multifid setae on the valvae of the male genitalia (Hodges 1998) (Fig. 10: *b s*). The subfamily has been placed in various gelechioid families, including Oecophoridae, Elachistidae, and most recently in the resurrected Depressariidae (Heikkilä et al. 2014) based on pupal morphology and weak molecular evidence. Depressariid pupae share lateral condyles between abdominal segments. Unlike most other depressariids, stenomatines have a gnathos that is apically smooth, not scobinate or spiculate.

*Antaeotricha* as a genus is diagnosed by the presence of a thumb-like process on the costa of the male valva (Fig. 14: *th*) and by the reduction of the anterior apophyses of the female ovipositor (Fig. 18: *a a*) (Duckworth 1964). Most species have white or brown wings with some contrasting maculation, usually the postmedial line, but often reduced to one or two spots on the discal cell. The newly described species has totally immaculate white wings.

The two previously published keys to *Antaeotricha* are based on wing pattern (Barnes and Busck 1920) or genitalia (Duckworth 1964). We provide two revised keys based on maculation and genitalia that treat pale-winged species of *Antaeotricha* and similar gelechioids.

## Materials and methods

Dissection of genitalia follows Robinson (1976): abdomens were macerated in hot 10% aqueous KOH, brushed clean in water, stained with Chlorazol Black, and slide-mounted in Euparal or stored in glycerin. Photographs were taken with 1) a Canon PowerShot Pro1 and 2) the Auto-montage Pro 5.01 system (Synoptics Ltd.) using a JVC digital camera and Leica Z16APO lens (FSCA). Habitus photographs were taken over standard 18% gray card under tungsten or fluorescent LED lights, and genitalia photographs were lit from above and below. Postprocessing was limited to 1) stacking images with Auto-Montage and 2) using Adobe Photoshop Elements 11 to standardize the gray (for habitus) or white balance (for slides) and the Auto Contrast function for some genitalia images. The map was made with Diva-GIS version 7.5.0.0 using a county-level shape file available from the website (www.diva-gis.org) and a CSV file of georeferenced decimal-degree coordinates.

Morphological terminology for genitalia follows Klots (1970) except where superseded by Kristensen (2003).

### Repository abbreviations are as follows

MVABS Archbold Biological Station (Lake Placid, FL, USA)

CMNH Carnegie Museum of Natural History (Pittsburgh, PA, USA)

CNC Canadian National Collection of Insects, Arachnids, and Nematodes (Ottawa, Canada)

FSCA Florida State Collection of Arthropods (Gainesville, FL, USA)

MEM Mississippi Entomological Museum (Starkville, MS, USA)

MGCL McGuire Center for Lepidoptera and Biodiversity, Florida Museum of Natural History (Gainesville, FL, USA)

NMNH National Museum of Natural History (Washington, D.C., USA)

TSDC Terhune S. Dickel Collection (Anthony, FL, USA)

## Systematics

### Antaeotricha floridella

Taxon classificationAnimaliaLepidopteraDepressariidae

Hayden & Dickel sp. n.

http://zoobank.org/A09EC2FE-9F0C-4CE7-A3C2-614278B9FD22

Figs 1–5 , 10–12 , 16–17

#### Type material.

**Holotype** ♂**. USA, Florida**: “FLORIDA: Marion Co. | Ocala National Forest | Forest Road 88 | 3.9 Miles SE of SR 316 | Longleaf Pine Sandhills | 14 OCT 2004 BL | TERHUNE S. DICKEL”, “FLMNH-MGCL | Specimen 164263” (FSCA). **Paratypes**: 1 ♂, same data as holotype (FSCA); 1 ♂: same data except 14 Sept. 2003 UV (FSCA); 1 ♂: same data except 21 Sept. 2003 UV (FSCA); 1 ♂: same data except 1 Oct. 2003 UV (FSCA); 1 ♂: same data except 20 July 2004 (FSCA); 1 ♂: same data except 18 Oct. 2004 MV/BL (FSCA); 4 ♂: same data except 20 OCT 2005 MV/BL (one MGCL 2404) (FSCA); 1 ♂, 1 ♀: FLORIDA: Marion Co. Ocala National Forest Forest Road 97 0.5 Miles S of SR 316 Sand Pine Scrub AUG 11 2007 MV TERHUNE S. DICKEL (FSCA); 2 ♂: same data except Aug. 17 2007 (FSCA); 5 ♂, 2 ♀: same data except 3.75 Miles SSE of SR 314 Sand Pine Scrub June 14 2005 MV/BL (FSCA); 2 ♀: Marion Co. Ocala Nat. Forest, 2 mi. S. of Juniper Sprs. 25-V-1993 Linwood C. Dow, [one] MGCL slide 2965 (MGCL); 3 ♂: Putnam Co., Welaka Forest Conservation Station, Site 4, live oak xeric hammock, 28–31-VII-1986, J.B. Heppner (FSCA); 1 ♀: same data except Site 5, slash pine-palmetto flatwoods (FSCA); 1 ♀: Putnam Co. IFAS Sta. Welaka 19 Sept ‘87 Dow (MGCL); 1 ♀: [Osceola Co.] Kissimmee, 24 Sept ‘83 L.C. Dow, MGCL slide 2964 (MGCL); 1 ♂, 2♀: Martin Co., Jonathan Dickinson State Park, 8–10 Aug. 1999, J.B. Heppner, MGCL slides 1679M, 1680F (FSCA); 1 ♂: Hillsborough Co., Tampa, USF Golf Course, bait trail, 4-V-1981, H.D. Baggett (FSCA); 1 ♂: Hillsborough Co., Tampa, USF Nature Preserve near Tampa campus, 5-X-1983, H.D. Baggett (FSCA); 1 ♂: Highlands Co., Archbold Biological Station, 10 mi. S. Lake Placid, UV light, 2-V-1975, J.B. Heppner (FSCA); 2 ♂: same data except 1-V-1975, (one) MGCL slide 1685M (FSCA); 1 ♂: same locality, 9-VII-1979, insect flight trap, H.V. Weems Jr. & Cathy W. Harris (FSCA); 3 ♂: Marion Co., Ocala National Forest, vic. Hopkin’s Prairie, 11–18-V-1979, Fairchild & Weems, (one) MGCL slide 1672M (FSCA); 1 ♂: same locality, 18-V-1979, insect flight trap, G.B. Fairchild (FSCA); 1 ♀: same data as previous except 23-V-1979, MGCL slide 1693F (FSCA); 1 ♂: Lake Co. SR 19, 5 miles S of Hwy 40, from larva on *Quercus geminata*, 15-IX-1989 (pupa 23-IX, adult 2-X-1989), D.H. Habeck and J. Gillmore, Habeck rearing A-5188 (FSCA); 6 ♂, 5 ♀: Putnam Co. near Hollister, larvae collected on “*Quercus minima*?”, 29-VIII-1984, D.H. Habeck, rearing A-3586 (FSCA); 1 ♀: Marion Co. 18 mi. E. of Lynne, 7-V-1981, adult 9-V-1981, host *Galactia regularis*, D.H. Habeck, rearing A-2790 (MGCL slide 1706) (FSCA); 1 ♂: FLA: Marion Co. 30 Oct. 2001 ONF Vargo (FSCA); 1 ♂: Highlands Co. Archbold Biological Station, June 3 1986, M.C. Minno, at UV light (MVABS); 1 ♂, 1 ♀: same data except June 4 1986 (MVABS); 1 ♂: same data except June 10 1986 (MVABS); 1 ♂: same data except July 13 1986 (MVABS); 1 ♂: same data except July 28 1986 (MVABS); 1 ♀: same data except July 20 1986 and “Reared ex larva in leaf & frass nest on *Quercus geminata*”, with pupal case (JEH genitalia slide 2777) (MVABS); 3 ♂: Lake Placid, 30 April 1964, R. W. Hodges (NMNH); 6 ♂: Lake Placid, Archbold Biol. Sta. 1-7 May 1964, R. W. Hodges, one each with USNM Genitalia Slide nos. 76253, 76323, 76324, 76325 (NMNH); 3 ♂, 3 ♀: same data except 8-15 May 1964, males not dissected, females USNM Genitalia Slide nos. 76319, 76320, 76321 (NMNH); 1 ♂: Dade City, October, Duckworth genitalia slide 102258-D, USNM slide no. 76305 (NMNH); 1 ♀: Citrus Co. Lecanto, 1 Oct. 1996, J. Glaser, J.E. Hayden slide no. 2752 (NMNH); 1 ♀: Highlands Co. Archbold Biol. Station 10 mi. S. Lake Placid 8-V-1975 at UV light J.B. Heppner (NMNH); 1 ♂: FLA., Highlands Co. Archbold Biol. Sta. Lake Placid 13 June, 1987 T.L. Schiefer; MEM 45,268; J.E. Hayden Slide No. 2979M (MEM); 1 ♂: FLA: Flagler Co. Pellicer Crk, 13 mi N of Bunnell. 1954 J. Bauer. C.M. Acc. 17023, 10.iv. (CMNH); 1 ♀: FLA: Flagler Co. Pellicer Crk, 13 mi N of Bunnell. 1954 J. Bauer. C.M. Acc. 17023, 21.vi. (CMNH); 1 ♀: Florida, Highlands Co., Lake Placid, Archbold Biological Station, W Jay Cottage, 27.1716°N 81.3493°W 21-VI-2006 at BL & MVL J.F. Landry & P.D.N. Hebert, slide MIC 6863, Barcode of Life #CNCLEP00025987 (CNC). Paratypes deposited in MVABS, CMNH, CNC, NMNH, and FSCA (FLMNH-MGCL Specimen nos. 164264–164315, 164327–164331).

#### Diagnosis.

Dorsally, the forewings of *Antaeotricha floridella* are immaculate white without any trace of a black spot at the distal end of the discal cell, and the hind wings are always pale gray. *Antaeotricha albulella* has one or two small black dots on the forewing at the distal end of the discal cell (Figs 7–9). A spot is usually visible under magnification even when worn, but some fresh specimens lack the spot entirely. The hind wings of *Antaeotricha albulella* are usually white, but some specimens have pale gray hind wings. *Antaeotricha osseella* (Walsingham, 1889) (Fig. 6) and *Antaeotricha unipunctella* (Clemens, 1863) are pale tan, ochreous or straw-colored with one or two black spots on the distal end of the forewing discal cell. The Western species *Antaeotricha thomasi* (Barnes & Busck, 1920) and *Antaeotricha utahensis* Ferris, 2012 are larger (forewing length ≥10 mm) and creamy white. *Antaeotricha utahensis* has a glossy sheen with small, scattered brown scales (Ferris 2012).

**Figures 1–9.**
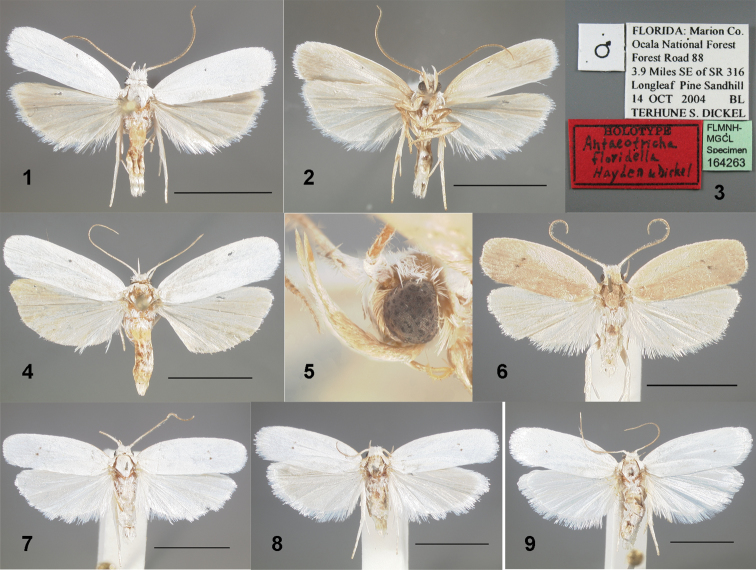
*Antaeotricha* spp. habitus. **1** *Antaeotricha floridella*, holotype male, dorsum **2** same, venter **3** labels of holotype **4** female, Marion Co. Florida (FSCA) **5** *Antaeotricha floridella*, Marion Co. Florida (T.S. Dickel Collection), lateral view of male head **6** *Antaeotricha osseella*, Putnam Co. Florida (FSCA) **7** *Antaeotricha albulella*, male, Alachua Co. Florida (FSCA) **8** *Antaeotricha albulella*, male with grayish hind wings, Suwannee Co. Florida (FSCA) **9** *Antaeotricha albulella*, female, Alachua Co. Florida (FSCA). Scale bars = 5 mm.

The most obvious genitalic difference between *Antaeotricha floridella* and *Antaeotricha albulella* is the shape of the gnathos. In *Antaeotricha floridella*, the lobes of the gnathos are flat, round-tipped, and close together, with the length of each lobe not longer than the common stalk. This is like the shape in *Antaeotricha osseella* and *Antaeotricha unipunctella*, but in *Antaeotricha albulella* (Figs 13, 15), the lobes of the gnathos are acute and widely spaced, with a U- or H-shaped embayment between them, and their common stalk is shorter than each lobe, almost non-existent. The gnathos of *Antaeotricha thomasi* and *Antaeotricha utahensis* is not bifid. The anellus of *Antaeotricha floridella* has two lobes on each side, and the dorsal (interior) lobe bears a few chaetae that are shorter than the lobe itself. In *Antaeotricha albulella* and *Antaeotricha osseella*, the interior lobes of the anellus bear more robust chaetae that are about as long as the lobe itself. Some specimens of *Antaeotricha albulella* have sub-apical chaetae; these are not present in *Antaeotricha floridella*, although some specimens have a fine sub-apical seta. The genitalia of *Antaeotricha floridella* are similar to those of *Antaeotricha unipunctella* depicted by Barnes and Busck (1920, Pl. XXIX fig. 3). Floridian specimens of *Antaeotricha unipunctella* have two anellar lobes rather than the four figured by Barnes and Busck (1920) and Duckworth (1964). *Antaeotricha thomasi* and *Antaeotricha utahensis* have no anellar lobes.

**Figures 10–19.**
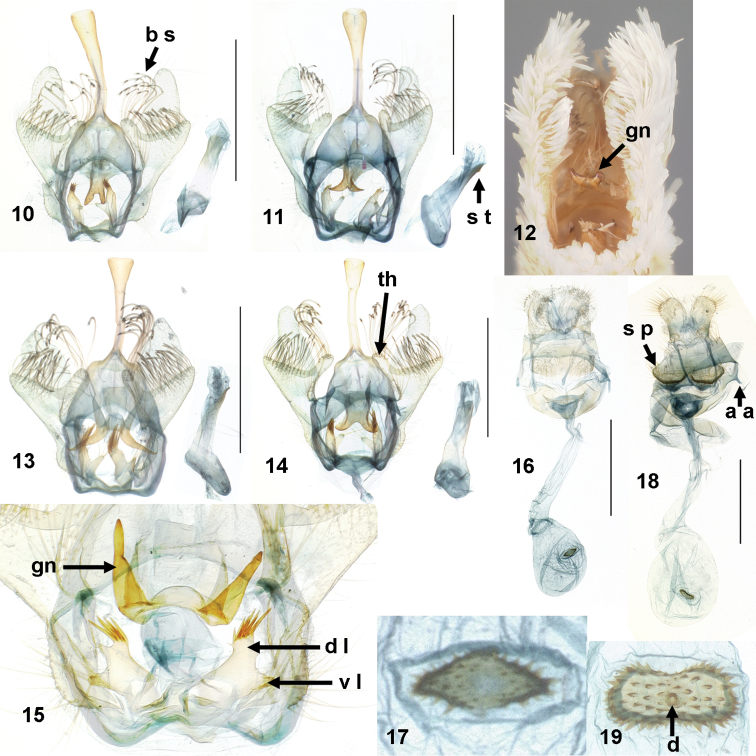
*Antaeotricha* spp. dissected genitalia. **10–15** (males): **10** *Antaeotricha floridella* male genitalic capsule and phallus (Marion Co. Florida, MGCL slide 1672) **11** *Antaeotricha floridella* (Martin Co. Florida, MGCL slide 1679) **12** *Antaeotricha floridella*, male genitalia in external view **13** *Antaeotricha albulella*, Sarasota Co. Florida (FSCA, MGCL slide 1736); 14. *Antaeotricha osseella*, Marion Co. Florida (FSCA, MGCL slide 1698) **15** *Antaeotricha albulella*, Levy Co. Florida, detail (FSCA, MGCL slide 1681) **16–19** (females): **16** *Antaeotricha floridella*, Martin Co. Florida (FSCA, MGCL slide 1680) **17** same as (**16**), detail of signum **18** *Antaeotricha albulella*, Escambia Co. Florida (FSCA, MGCL slide 1691) **19** *Antaeotricha osseella*, New Mexico, Otero Co. detail of signum (FSCA, MGCL slide 1721). *a a*, anterior apophysis (reduced); *b s*, bifid setae; *d*, central denticle of signum; *d l*, dorsal (interior) lobe of annellus; *gn*, gnathos; *s p*, setose pads of sternum VIII; *s t*, subapical tooth of phallus; *th*, thumb-like process of valva; *v l*, ventral (exterior) lobe of annellus. Scale bars = 1 mm.

In the female genitalia of *Antaeotricha floridella*, sternite VIII consists of two flat setose pads that are broadly joined mesally and not bordered by pockets on the anterior margin. In contrast, these pads in *Antaeotricha albulella* distinctly protrude out of the plane of the sternite and are partly divided by an anterior emargination (Fig. 18). There is a shallow pocket in the membrane between each protrusion and SVIII. These protrusions are diagnostic for *Antaeotricha albulella* and are obvious in the undissected lectotype specimen. The ductus bursae of *Antaeotricha floridella* is slightly longer than the corpus bursae, whereas *Antaeotricha albulella* has a ductus bursae as long as the corpus bursae. The signum of *Antaeotricha floridella* varies in shape but is generally rhombiform or trapezoidal, being at least as wide or wider mesally than at either end, and it lacks a central denticle. In *Antaeotricha albulella*, *Antaeotricha osseella*, and *Antaeotricha unipunctella*, the signum is more or less arachiform (peanut-shaped), being mesally constricted or at least linear in *Antaeotricha unipunctella*. In those species, the signum frequently has a central denticle in addition to scattered lateral denticles, which is especially well-developed in *Antaeotricha osseella* (Fig. 19). The central denticle is absent in *Antaeotricha floridella*. The signum of *Antaeotricha thomasi* and *Antaeotricha utahensis* is differently shaped (cruciform in the former and hexagonal in the latter).

#### Description.

*Head* (Fig. 5). Vertex white; frons white and laterally pale brown. Labial palpi with second segment laterally pale brown, mesally white; apical segment all white. Ocelli absent. Antenna white on scape, pedicel, and basal flagellomere, distal flagellomeres brown; male sensilla as long as width of flagellum; female sensilla short.

*Thorax.* Legs pale brown anteriad and laterally; mesally white. Spurs 0-2-4, inner twice length of outer.

*Wings* (Figs 1–2, 4). Mean forewing length of males: 6.6 mm, range 6.0–7.0 mm (n = 26); of females: 7.6 mm, range 6.5–8.5 mm (n = 15). Forewings with anterior and posterior margins parallel. Eleven veins arising separately from cell; Sc, R_1_, Rs_1–4_, M_1–3_ present; CuA_1_ and CuA_2_ from common point at corner of cell; CuP tubular in distal 2/5; 1A+2A forked at base. Forewings dorsally matte white with no trace of black discal spots or other maculation, or if greased, pale lemon yellow; costa proximally pale brown, distally white. Ventral side pale grayish yellow. Hind wing venation: Sc+R_1_ present; Rs_1_ stalked with M_1_ 1/4 their length; M_2_ present; M_3_ and CuA_1_ from common point at corner of cell; CuA_2_ from well before end of cell; CuP, 1A+2A, 3A present. Hind wings pale gray on both sides, fringe white; male with tuft of short hairlike scales from base of discal cell on dorsal side. Male retinacular hook present; female frenulum double or triple.

*Abdomen.* Uniformly white, without androconia or otherwise modified scales.

*Male genitalia* (Figs 10–12). Uncus slender, distally bulbous and truncate. Gnathos with two lobes, flattened and broadly pointed, lobes as long as common stalk, set closely. Vinculum broadly concave ventrally. Distal half of valva narrowly rounded and membranous; medio-central extension of valva (“harpe” of Duckworth 1964) mitten-shaped, with “thumb” on costal half parallel-sided, twice as long as wide, bearing long, curved, bifid setae, and with lateral half broadly triangular, with short, straight, bifid setae. A narrow, setiferous ridge present basal of thumb. Each half of anellus with two lobes: ventral (exterior) lobe with long setae, dorsal (interior) lobe with a few short chaetae restricted to apex, no longer than the lobe. Phallus without cornuti; with (specimens from southern Florida) or without low subapical tooth.

*Female genitalia* (Figs 16–17). Papillae anales rounded, setose. Apophyses anteriores elongate, extended to posterior margin of sternum VII. Apophyses anteriores short. Sternum VIII with two square setose pads, not or only slightly projected, joined mesally the entire length but not medially setose. AVII–AVIII membrane without concavities. Ostium bursae in middle of sternum VII. Lamella antevaginalis a narrow, curved band. Lamella postvaginalis with a broad, triangular extension covering ostium. Ductus bursae as long or longer than corpus bursae, not sclerotized, leading straight into corpus bursae or with a half-twist. Ductus seminalis from near posterior end of ductus bursae. Corpus bursae pyriform. One signum (Fig. 17) situated halfway along corpus bursae, bulged inward, roughly rhombiform with transverse axis the longer; shape variably ovoid, rhomboid, or with anterior side slightly more acute than posterior; denticles present on each half, without central denticles along mesal suture or in center.

#### Etymology.

The specific epithet is an adjective derived from the state of Florida, diminutive like congeners.

#### Distribution.

**Figure 20.**
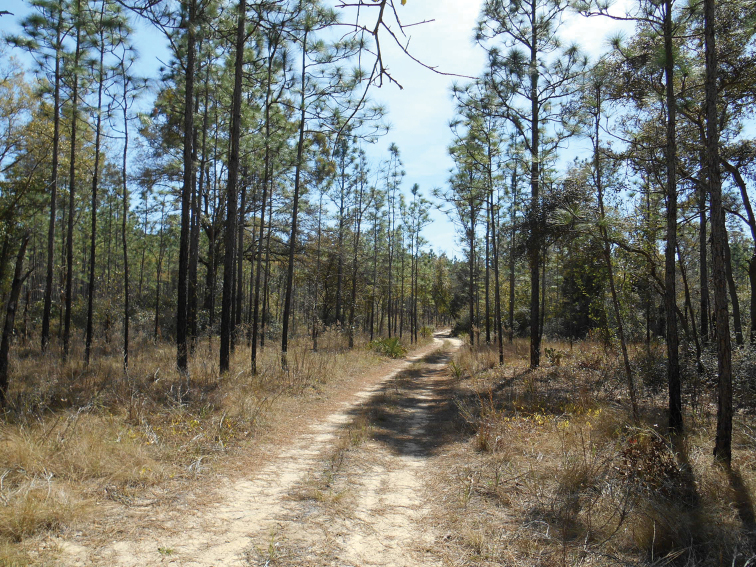
Type locality south of Lake Kerr in Ocala National Forest, Florida (29.3320°N, 81.7807°W). Sandhill dominated by pines and understory oaks.

The type locality is in Ocala National Forest (Marion County, Florida, USA) south of Lake Kerr in sandhills dominated by longleaf pine (*Pinus palustris* Mill.) (Fig. 20). The known distribution is peninsular Florida, including ten counties: Citrus, Flagler, Highlands, Hillsborough, Lake, Marion, Martin, Osceola, Pasco, and Putnam.

**Figure 21.**
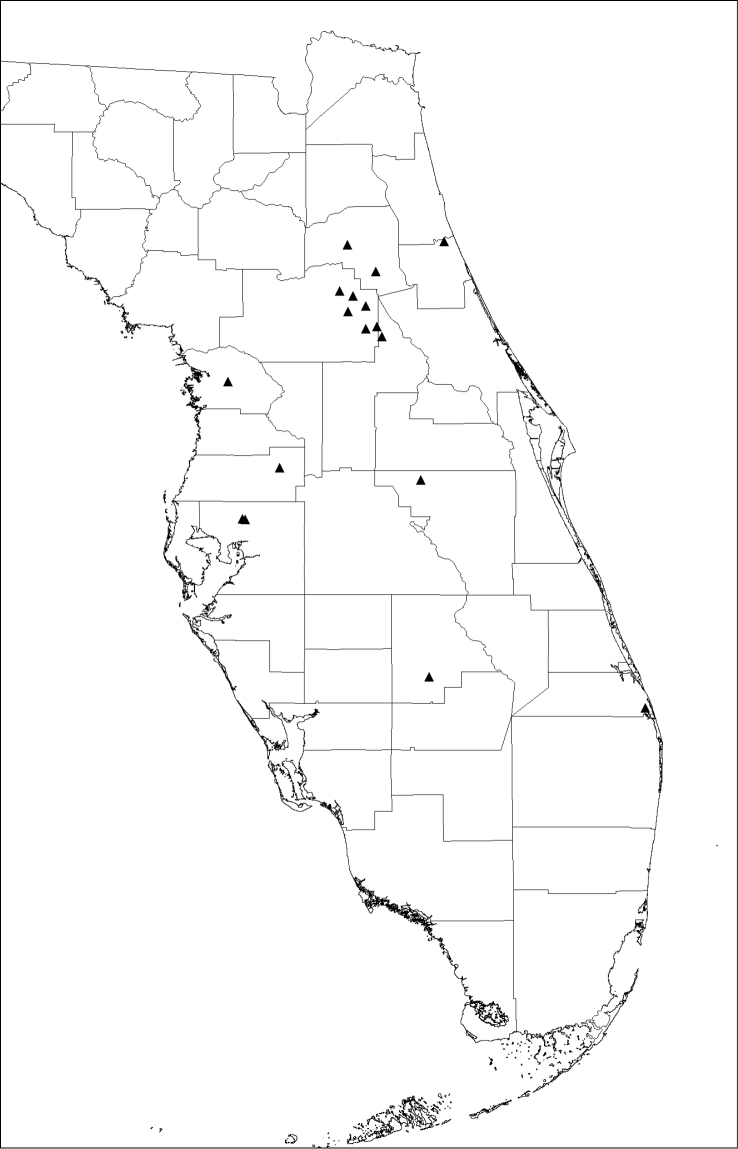
Distribution of *Antaeotricha floridella* in Florida, USA (triangles).

#### Ecology.

*Antaeotricha floridella* has been reared on leaves of *Quercus geminata* Small (sand live oak) and *Quercus minima* (Sarg.) (dwarf live oak; identified with “?”). D.H. Habeck reared a specimen on *Galactia regularis* (L.) (downy milkpea). Adults have been collected April 10–October 30.

## Discussion

Becker (1981) synonymized *Antaeotricha vestalis* (Zeller, 1873) with *Antaeotricha albulella* (Walker, 1864), and he remarked that the lectotype of *Antaeotricha albulella* and the available specimens of *Antaeotricha vestalis* all had black discal spots. Zeller (1873) stated that the type of *Cryptolechia vestalis* had white forewings “*ohne jede Zeichnung*” (without any marking), but the hind wings are white like the forewings. The lectotype specimen, deposited in the Museum of Comparative Zoology (Cambridge, MA, USA) is a female from Texas. Its forewings have faint black discal spots, and sternum VIII has the typical setose protuberances, which are visible without dissection.

*Antaeotricha floridella* is described as a new species because no other white species with similar genitalia were described by Meyrick (Clarke 1955), Walsingham (1909–1915), Walker (1864), Zeller (1854, 1855, 1873, 1877) or other authors. The West Indian *Antaeotricha* fauna is depauperate, with only three species (Duckworth 1969). Descriptions and illustrations of all species currently placed in *Antaeotricha* were examined, a task greatly facilitated by the illustration of Meyrick’s numerous species by Clarke (1955) and by the concentration of other descriptions among a few authors (e.g. Walker 1864; Walsingham 1909–1915; Busck 1911, 1920; Zeller 1854, 1877; see Becker 1984 for complete list). Almost all species are described as having some forewing maculation, and those without are some shade of brown.

The new species keys to couplet 8 in Barnes and Busck (1920: 238), requiring a new line, “forewings pure white without discal spots.” In the key of Duckworth (1964: 27), males run to couplet 13 (*Antaeotricha unipunctella* or *Antaeotricha vestalis*), and females run to *Antaeotricha osseella* or *Antaeotricha unipunctella*. New keys are provided below that include *Antaeotricha floridella* and another species described after 1964.

Duckworth’s (1964) key is inaccurate as it pertains to the small, unicolorous species. In couplet 12 of the key, the “[g]nathos divided into two lobes at tip” could apply equally to *Antaeotricha osseella* and *Antaeotricha decorosella* (Busck, 1908 a) as well as to *Antaeotricha unipunctella* and *Antaeotricha albulella*; he correctly noted the “notched” condition of the gnathos in the species accounts. In couplet 14, the low subapical spine or tooth of the phallus (Fig. 11: *s t*) is stated to be present in *Antaeotricha osseella* and absent in *Antaeotricha decorosella*. In our dissections, the spine in *Antaeotricha osseella* varies from sharply angulate to a low hump to entirely absent. In a specimen that appears to represent *Antaeotricha decorosella* (MGCL slide 2166), a low rise is present. This specimen has many robust chaetae on the apical two thirds of the anellar lobe, which is diagnostic for male *Antaeotricha decorosella*. Furthermore, the variation of the spine is as follows: *Antaeotricha unipunctella*, absent or a low serrate ridge; *Antaeotricha albulella*, a very low expansion but not an angulate tooth; *Antaeotricha floridella*, present or absent. This variable character should be avoided for separating species.

*Antaeotricha albulella*, *Antaeotricha osseella*, and *Antaeotricha floridella* adults are active at the same time and location. John B. Heppner has caught all three species at the Welaka Forest Conservation Station, 28–31 July 1986. A male specimen each of *Antaeotricha albulella* and *Antaeotricha floridella* were collected at Pellicer Creek (Flagler Co.) on 10 April 1954 (CMNH). The road numbers in Ocala National Forest changed in 2008: Forest Road 88 is now 11, and 97 is now 09.

*Antaeotricha floridella* is known to feed on two species of oak: *Quercus geminata* and possibly *Quercus minima*. The latter species may be misidentified, since it resembles juvenile or rhizomatous forms of other oaks (Nixon 1993). Host plants of *Antaeotricha albulella*, recorded from pinned specimens donated to the FSCA by D.H. Habeck, include *Quercus laevis* Walter (turkey oak), *Quercus nigra* L. (water oak) (with feeding habits “galls” and “leaf tier”), *Quercus incana* W. Bartram (bluejack oak), *Quercus myrtifolia* Willd. (myrtle oak), *Quercus inopina* Ashe (scrub oak), and *Quercus chapmanii* Sarg. (Chapman’s oak). The plant voucher specimens could not be located, so their identifications could not be verified.

Two plant communities in Ocala National Forest in Marion and Putnam counties, the sandhill and sand pine communities, have been collected extensively by TSD over the past several years using both mercury vapor light and ultraviolet light. *Antaeotricha floridella* occurs in both plant communities, as well as in strict scrub habitat with minimal canopy not surveyed by TSD. In the sandhill community, the common species of pine is *Pinus palustris* Miller (longleaf pine), and the common species of oak is *Quercus laevis*. The leaves of turkey oak are deciduous, mostly falling in September and October, with a few leaves remaining on the trees during the winter. New foliage begins to appear in March and April. In the sand pine community, the predominant pine is *Pinus clausa* Chapman ex Engelmann (sand pine), and the common oak is *Quercus myrtifolia*. In Ocala National Forest, myrtle oak tends to be a thicket-forming shrub. Leaves are “tardily deciduous,” meaning that a few leaves fall during the winter months, but the majority of leaf fall occurs during late February and March, just as the trees begin to flower and new leaves develop. *Quercus geminata* is also tardily deciduous (Godfrey 1988). The type locality of *Antaeotricha floridella* has *Quercus geminata*, *Quercus laevis*, and *Quercus hemisphaerica* in abundance, and *Quercus myrtifolia* and *Quercus nigra* in small numbers.

Extensive collecting in a large mesic forest near Anthony (Marion County, Florida) by TSD with mercury vapor and ultraviolet lights and sugar bait has failed to produce any specimens of *Antaeotricha floridella*. This forest has large numbers of three species of oaks: *Quercus virginiana* Miller (live oak), *Quercus hemisphaerica* Bartr. ex Willd. (laurel oak), and *Quercus nigra* (water oak). The leaves of all of these species are tardily deciduous with primary leaf fall occurring in late February and March just prior to flowering and new leaf growth.

With minimal host information, it is open to question whether *Antaeotricha floridella* is monophagous on *Quercus geminata*, oligophagous on oaks with overwintering foliage, or has more hosts. Sand live oak occurs in both plant communities and others in Florida. It occurs on the southeastern coastal plain from Virginia to Mississippi (Godfrey 1988), so the plant’s distribution cannot explain the moth’s restriction to peninsular Florida. On the other hand, a broader host range would predict occurrence in non-xeric habitats. The phenology of the immature stages is unknown, in particular of the overwintering stages. It is not obvious that *Antaeotricha floridella* has adaptations to abiotic characteristics of xeric habitats, so affinity for some host is assumed.

A partly historical explanation for the peninsular distribution may be isolated evolution in habitat islands of sandhill and scrub (Webb 1990). Few Lepidoptera are known to be restricted to those habitats and also endemic to Florida. One moth species is known to be endemic to Florida rosemary scrub, a geometrid that feeds on *Ceratiola ericoides* Michx. (Deyrup 1989). Kons and Borth (2006) list 51 species of Lepidoptera that, based on preliminary evidence, may be dependent on the first kind of plant community (turkey oak-longleaf pine sandhills), but the majority of these also occur in similar habitats outside peninsular Florida. Comparable data are lacking for sand pine communities. Although most specimens of *Antaeotricha floridella* were collected in longleaf pine sandhills or sand pine scrub, the specimens from Jonathan Dickinson State Park and Archbold Biological Station may have been collected in other kinds of scrub habitat (Myers 1990). It has not been found in collections (MEM, FSCA) from the ecologically similar Ohoopee Dunes in Georgia, although *Antaeotricha albulella* occurs there. Kons and Borth (2006) discuss in depth the methods and caveats of associating species with xeric habitats based mainly on adult specimens, and they emphasize the need to test hypotheses of habitat dependence with further collection data.

The *Antaeotricha albulella* group (including *Antaeotricha osseella*, *Antaeotricha unipunctella*, and *Antaeotricha decorosella*) is probably a recent radiation, with *Antaeotricha floridella* as a peninsular vicariant. It is not simply a peripheral isolate of *Antaeotricha albulella*, because it lacks the autapomorphies of the latter species (the broad gnathos and prominent SVIII pads). Preliminary genetic data corroborates the species’ distinct status. A specimen of *Antaeotricha floridella* in the CNC, dissected by J.-F. Landry, has a slightly greater percentage distance than intraspecific clusters of *Antaeotricha albulella* based on mtCO1 (J.-F. Landry, pers. comm. 2014). The sequence data are available at: http://www.boldsystems.org/index.php/Public_RecordView?processid=MNAB391-07. Study of more genetic data should be useful to clarify the *Antaeotricha albulella* group. All species and populations should be sampled and the data analyzed with character-based phylogenetic methods to discover diagnostic apomorphies. Collection of *Antaeotricha* specimens across known phylogeographic discontinuities in North Central Florida and the Panhandle could demarcate the northern limit of the distribution of *Antaeotricha floridella* (Soltis et al. 2006).

**Additional species examined.** Dissected specimens of several other species were examined to construct the keys, except *Gonioterma crambitella* (Walsingham, 1889), figured in Duckworth (1964). Unless otherwise indicated, dissection slides are assigned MGCL slide numbers and deposited in FSCA.

*Antaeotricha albulella*: FL, Collier Co. Fakahatchee Strand, MGCL 1953♂; FL, Collier Co., USNM slide 76303♂ (NMNH); FL, Duval Co., USNM slide 76306♂ (NMNH); FL, Escambia Co., MGCL 1691♀; FL, Highlands Co., MGCL 1699♀ (FSCA), USNM slides 76318♀, 76252♀ (NMNH); FL, Hillsborough Co., MGCL 1686♂; FL, Lee Co., USNM slide 76304♂ (NMNH); FL, Levy Co., MGCL 1681♂; FL, Miami-Dade Co., MGCL 495♂ (FSCA), USNM slides 76302♀, 135307♂ (NMNH); FL, Polk Co., USNM slide 76301♂ (NMNH); FL, Putnam Co., MGCL 1697♂; FL, Sarasota Co., MGCL 1736♂; FL, Volusia Co., USNM slide 135306♂ (NMNH); GA, Emanuel Co. Ohoopee Dunes, JEH 2970♂ (MEM); LA, St. John Parish, MGCL 1671♀; LA, St. John Parish, MGCL 1747♂; MD, Kent Co., JEH 2756♂ (NMNH); MD, Kent Co., JEH 2765♀ (NMNH); NC, Craven Co., MGCL 1670♂; NC, Craven Co., MGCL 1793♀; TX, Anderson Co., JEH 2753♂ (NMNH); TX, Anderson Co., JEH 2754♀ (NMNH); VA, Virginia Beach Co., JEH 2775♂ (NMNH); [no locality], USNM slide 135305♂ (NMNH).

*Antaeotricha arizonensis* Ferris, 2010: AZ, Cochise Co., MGCL 1733♂; AZ, Cochise Co., MGCL 1734♀.

*Antaeotricha decorosella*: MO, Benton Co., MGCL 2166♂ (MGCL).

*Antaeotricha furcata* (Walsingham, 1889): AZ, Gila Co., MGCL 1735♂; TX, Jeff Davis Co., MGCL 2074♀.

*Antaeotricha fuscorectangulata* Duckworth, 1964: AZ, Cochise Co., MGCL 1728♂.

*Antaeotricha haesitans* (Walsingham, 1912): TX, Hidalgo Co., MGCL 2065♂.

*Antaeotricha humilis* (Zeller, 1855): FL, Alachua Co., MGCL 1677♂; FL, Marion Co., MGCL 1678♀.

*Antaeotricha irene* (Barnes and Busck, 1920): TX, Hidalgo Co., MGCL 2066♂.

*Antaeotricha leucillana* (Zeller, 1854): FL, Alachua Co., MGCL 1689♂; FL, Alachua Co., MGCL 1690♀; ME, Waldo Co., MGCL 2076♂.

*Antaeotricha lindseyi* (Barnes and Busck, 1920): AZ, Cochise Co., MGCL 2075♂.

*Antaeotricha manzanitae* Keifer, 1937: CA, El Dorado Co., MGCL 1731♂ (MGCL); CA, El Dorado Co., MGCL 1732♀ (MGCL).

*Antaeotricha osseella*: FL, Alachua Co., MGCL 2077F, 2966F, 2967♂; FL, Escambia Co., MGCL 1676♀; FL, Highlands Co., MGCL 1675♂; FL, Marion Co., MGCL 1698♂, 2400♂; FL, Putnam Co., MGCL 1713♂; MO, Carter Co, MGCL 2164♂ (MGCL); MO, Carter Co., MGCL 2165♀ (MGCL); NC, Craven Co., MGCL 1711♂; NM, Otero Co., MGCL 1721♀.

*Antaeotricha schlaegeri* (Zeller, 1854): CANADA, Nova Scotia, MGCL 2375♂; USA, AZ, Santa Cruz Co., MGCL 1702♀; FL, Alachua Co., MGCL 1687♂, 1688♀; MA, Plymouth Co., MGCL 2365♂; MA, Plymouth Co., MGCL 2366♀; MO, Barry Co., MGCL 2370♀; MO, Clay Co., MGCL 2367♂; NC, Craven Co., MGCL 2373♂; TN, Sullivan Co., MGCL 2374♂.

*Antaeotricha unipunctella*: FL, Escambia Co., MGCL 1714♂; FL, Hernando Co., MGCL 2992♀; FL, Highlands Co., MGCL 2078♀, 2399♂; FL, Manatee Co., MGCL 1673♂, 2167♀; FL, Marion Co., MGCL 1674♀, 1712♂.

*Antaeotricha utahensis*: AZ, Cochise Co., MGCL 1703♂; NM, Grant Co., MGCL 1720♂.

*Autosticha kyotensis* (Matsumura, 1931): FL, Santa Rosa Co., MGCL 485♂, 486♀; FL, Gainesville, MGCL 487♂.

*Durrantia piperatella* (Zeller, 1873): OK, Latimer Co., MGCL 1727♂; TX, Brewster Co., JEH 2761♂, 2762♀ (NMNH).

*Gonioterma mistrella* (Busck, 1907): MO, Barton Co., MGCL 1695♂; MO, Newton Co., MGCL 1696♀.

*Inga cretacea* (Zeller, 1873): MO, Barry Co., MGCL 2062♂; AR, Washington Co., MGCL 2063♀.

*Pseuderotis obiterella* (Busck, 1908 b): NC, Craven Co., MGCL 1726♂.

### Keys to species

The following keys apply only to taxa with white or pale-colored wings (yellowish, pale orange, or beige) that are effectively concolorous. Species of *Antaeotricha* that have a dark shade on the the forewing posterior margin are excluded. Other stenomatines (*Gonioterma* Walsingham) and Oecophoridae that have similarly concolorous wings are included.

### Key based on wing pattern

**Table:** 

1	Forewing terminal spots present	**other Gelechioidea, including *Durrantia piperatella*, *Pseuderotis obiterella*, *Autosticha kyotensis***
–	Forewing terminal spots absent	**2**
2	Forewing transverse lines present	**3**
–	Forewing transverse lines absent	**4**
3	Forewing pale yellow	***Antaeotricha haesitans***
–	Forewing white, usually with scattered black scales	***Inga cretacea***
4	General color tan, beige, or yellow-orange	**5**
–	General color white or cream, at most pale yellow	**7**
5	Forewing grayish tan and distally truncate; forewing with 1 spot at distal end of cell, another spot 2/5 along anal fold; hind wing often much darker than forewing	***Gonioterma mistrella***
–	Forewing color variably tan, beige, orange, distally rounded in shape; forewing with one or two spots at distal end of cell but without spot on anal fold; hind wing never much darker than forewing	**6**
6	Hind wing white or pale yellow	***Antaeotricha unipunctella***
–	Hind wing pale tan or whitish fuscous	***Antaeotricha osseella*, *Antaeotricha decorosella***
7	Forewing with scattered fuscous scales (may be microscopic), not including discal spots	**8**
–	Forewing without fuscous scales except, at most, discal spot(s)	**9**
8	Forewing narrower (aspect ratio 3.67)	***Antaeotricha utahensis***
–	Forewing broader (aspect ratio 2.85)	***Gonioterma crambitella***
9	Wings white with one or two gray or black spots on discal cell (occasionally worn); hind wings usually white, rarely pale gray; eastern Nearctic (New Jersey to central Texas)	***Antaeotricha albulella***
–	Wings pale yellowish white with one dark spot on discal cell; hind wings same color; New Mexico and Arizona	***Antaeotricha thomasi***
–	Forewings white without discal spots; hind wings always pale gray; peninsular Florida	***Antaeotricha floridella***

### Key based on male and female genitalia (applicable only to pale-winged Stenomatinae and similar Gelechioidea in the Nearctic region)

**Table:** 

1	Valvae without bifurcate setae; either signum with two long posterolateral arms (*Autosticha*) or ovipositor elongate (membrane connecting SVIII and papillae anales at least twice length of SVIII)	**Oecophoridae, Autostichidae**
–	Valvae with apically bifurcate setae; signum without long arms and ovipositor not elongate (membrane connecting SVIII and papillae anales at most the same length as SVIII)	**2**
2	Valva without thumb-like process; apophyses anteriores elongate (*Gonioterma*)	**3**
–	Valva with thumb-like process; apophyses anteriores reduced (*Antaeotricha*)	**4**
3	Phallus large and flared apically; cornuti in two clusters; lobes of anellus triangular with round lateral margins; female with one signum	***Gonioterma mistrella***
–	Phallus small and broad; cornuti in one cluster; lobes of anellus very elongate and slender; female with two signa	***Gonioterma crambitella***
4	Uncus divided, at least in apical half; signum transverse (ovate or bilobate)	**5**
–	Uncus entire; signum shaped otherwise or absent	**9**
5	Uncus deeply divided to base, appearing as two widely separated, pointed processes; sterigma (abdominal sternite VIII) with setose lateral areas pronounced as bumps	***Antaeotricha albulella***
–	Uncus divided to half its length or less, with processes apically flattened and round-edged; sterigma with lateral setose areas flat or barely raised	**6**
6	Anellus with two lobes on each side, the interior lobe bearing many robust chaetae on apex and extended down the side; signum arachiform (peanut-shaped), distinctly narrower in middle than laterally	**7**
–	Anellus with one or two lobes; if two, robust chaetae concentrated apically, or phallus without single apical tooth; signum rhombiform, trapezoidal, or rod-shaped, but middle not distinctly narrower than either end	**8**
7	Robust chaetae near apex of interior anellar lobe; phallus usually with apicoventral tooth; genital plate anterior of ostium with elevated extension posterior of ostium	***Antaeotricha osseella***
–	Chaetae more extensive, on apical 1/3 to 2/3 of anellar lobe; phallus without tooth; genital plate without elevated area posterior of ostium	***Antaeotricha decorosella***
8	Anellus with two lobes on each side, the interior one bearing a few small chaetae at far apex, often fused with lobe; phallus with apical tooth; signum variably rhombiform or trapezoidal, without central denticle, only lateral denticles present	***Antaeotricha floridella***
–	Anellus with one or two lobes, if two, the interior lobe with larger chaetae on more than the far apex; phallus either without apical tooth or with elongate serrate ridge; signum usually rod-shaped, with one central denticle in addition to many lateral denticles	***Antaeotricha unipunctella***
9	True cornuti absent but phallus with three pairs of lateral processes; signum absent	***Antaeotricha haesitans***
–	Cornuti present on vesica; signum present	**10**
10	Uncus apically bifid; signum with four truncate points	***Antaeotricha thomasi***
–	Uncus apically pointed; signum with six truncate points	***Antaeotricha utahensis***

## Supplementary Material

XML Treatment for Antaeotricha floridella
